# Surgical margins in head and neck squamous cell carcinoma: A narrative review

**DOI:** 10.1097/JS9.0000000000001306

**Published:** 2024-03-11

**Authors:** Yang Chen, Nian-Nian Zhong, Lei-Ming Cao, Bing Liu, Lin-Lin Bu

**Affiliations:** a State Key Laboratory of Oral & Maxillofacial Reconstruction and Regeneration, Key Laboratory of Oral Biomedicine Ministry of Education, Hubei Key Laboratory of Stomatology; b Department of Oral & Maxillofacial – Head Neck Oncology, School & Hospital of Stomatology, Wuhan University, Wuhan, People’s Republic of China

**Keywords:** assessment method, head and neck squamous cell carcinoma, neoadjuvant therapy, surgery, surgical margin

## Abstract

Head and neck squamous cell carcinoma (HNSCC), a prevalent and frequently recurring malignancy, often necessitates surgical intervention. The surgical margin (SM) plays a pivotal role in determining the postoperative treatment strategy and prognostic evaluation of HNSCC. Nonetheless, the process of clinical appraisal and assessment of the SMs remains a complex and indeterminate endeavor, thereby leading to potential difficulties for surgeons in defining the extent of resection. In this regard, we undertake a comprehensive review of the suggested surgical distance in varying circumstances, diverse methods of margin evaluation, and the delicate balance that must be maintained between tissue resection and preservation in head and neck surgical procedures. This review is intended to provide surgeons with pragmatic guidance in selecting the most suitable resection techniques, and in improving patients’ quality of life by achieving optimal functional and aesthetic restoration.

## Graphical abstract

**Figure:**
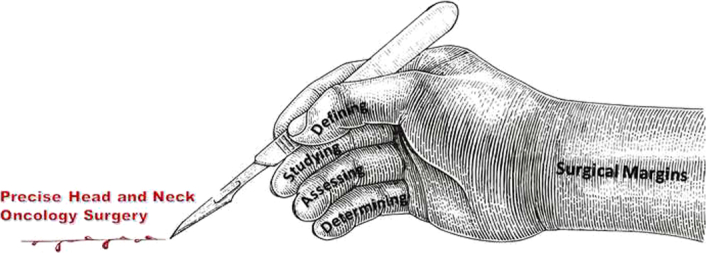

## Introduction

Highlights

- We have conducted a comprehensive review of the cut-off value and the factors that influence the surgical margins (SMs) in head and neck squamous cell carcinoma.
- This presentation explores SMs in a range of clinical scenarios, with a special focus on the differing interpretations of the SMs following neoadjuvant therapy, a topic that deeply concerns surgeons.
- We discuss the capabilities and potential of artificial intelligence when combined with assessment techniques to distinguish head and neck squamous cell carcinoma margins.
- Furthermore, we suggest that the scope of surgical resection should include the feasibility of reconstruction rather than merely aiming for minimal surgical resection.

Head and neck cancers (HNCs) rank as the seventh most prevalent form of cancer globally, with a reported 890 000 new instances and 450 000 deaths in 2018^1^. The most frequent histological subtype is head and neck squamous cell carcinomas (HNSCCs), accounting for more than 90% of all cases^2,3^. These predominantly originate from the mucosal epithelium within the oral cavity, pharynx, larynx, and sinonasal tract^4^.

Multidisciplinary treatment, typically comprising surgery, radiotherapy, and systemic therapy, is required for most HNSCC cases. Surgery is an essential therapeutic strategy for HNSCC across T1–T4a, and the surgical margin (SM) is one of the most crucial aspects that surgeons can manipulate to enhance prognosis^4,5^. Achieving clear margins/R0 resection during the initial surgical intervention can safeguard patients against repeat surgery and adjuvant therapies or even escalate such treatments^6–9^. However, increasing the likelihood of clear margins through the excessive removal of healthy tissue can lead to larger defects and decreased quality of life for patients^10^. Consequently, the therapeutic objective for HNSCC is to strike a balance between both sides of the ‘seesaw’ – a favorable prognosis and minimal tissue defect, underscoring the importance of determining an appropriate SM.

In an effort to more accurately define tumor margins and execute the most fitting resection, our review centers on the recommended SM under different conditions, the variety of margin assessment methodologies, and strategies to maintain equilibrium between tissue resection and preservation during head and neck surgeries. In this review, ‘HNSCC’ primarily denotes squamous cell carcinoma (SCC) of the oral cavity (OSCC), oropharyngeal (OPSCC), larynx (LSCC), nasopharyngeal (NPC), and hypopharyngeal (HPSCC). SCC of the paranasal sinuses and nasal cavities is not included in this discussion due to the dearth of available reports on their SMs.

## A brief history of head and neck oncologic surgery

The inception of head and neck oncologic surgery can be dated back to 30 AD^11^. Despite its extended history, the field witnessed a period of accelerated development from the 19th century onward. From the mid-19th to the conclusion of the 20th century, the paradigm of head and neck oncologic surgery experienced approximately three phases: the ‘simple tumor organ resection’ phase, the ‘extensive radical resection’ phase (involving continuous *en bloc* resection of the primary tumor and cervical lymph nodes), and the ‘function preservation surgery’ phase (*en bloc* resection of the primary tumor coupled with modified radical neck dissection or functional neck dissection)^11–13^.

Transitioning into the 21st century, the term ‘minimally invasive’ has become a guiding principle in HNC surgery. This concept, initially introduced by Payne *et al*. in 1985^14^, had begun to surface as a prominent trend by the mid to late 20th century. Accompanied by swift advancements in technology, including endoscopy, laser surgery, and robot-assisted surgical systems, along with a variety of imaging tools, the pursuit of minimally invasive surgery perseveres. The objective is to conserve the patient’s anatomy and function as much as feasible while ensuring survival^15,16^.

## Surgical indications and contraindications

Prior to deliberating on the SMs of HNSCC, it is crucial to evaluate the feasibility of tumor removal^17^. Although surgery is the traditional treatment modality for HNSCC, performing radical resections without due caution on some locally advanced HNSCC (LAHNSCC) cases could result in irreversible loss of tissue and function^18^. Tumors that cannot be technically excised, or those predicted to result in unacceptably detrimental functional consequences postoperatively, can be designated as ‘unresectable’ or ‘inoperable’. These usually fall into the T4b category, which includes tumors with extensive involvement of structures such as the skull base, mediastinal structures, prevertebral fascia, cervical vertebrae, brachial plexus, or critical head and neck vasculature^19,20^. NPC, conversely, is usually deemed unresectable, and hence, radiotherapy emerges as the primary therapeutic approach^20^. Importantly, the designation of a tumor as ‘unresectable’ is not definitive but rather suggests that if surgical intervention is pursued as the primary management strategy, the prospects of achieving R0 resection, functional reconstruction, or favorable local control (LC), even with adjuvant therapy, may be dim^17^. As such, for these ‘unresectable’ LAHNSCC, chemoradiotherapy (CRT) is typically recommended^21^. However, given an adequate resection distance, patients with pathologically classified T4b OSCC may also exhibit favorable prognoses post-surgery. This observation may be attributed to the potential difficulty in strictly distinguishing between T4a and T4b stages^22,23^.

## Surgical margins in head and neck squamous cell carcinoma

Surgery currently holds its place as the most efficacious treatment modality for HNSCC^24^. Not only is the SM an independent prognostic determinant for patients with HNSCC, but it is also the only factor over which surgeons have significant influence^25^. In the majority of cases involving superficial tumors, surgeons delineate the extent of the tumor based on visual observation, tactile perception, and professional experience^26^. It is critical to remember that microscopic tumor cells often extend beyond visible lesions, prompting surgeons to excise the visible tumor in conjunction with a portion of the surrounding ‘normal tissue’, rather than confining the resection to the visible tumor margin^27^. Therefore, identifying the proper location of safe SM across varying clinical scenarios is a pivotal matter in order to ensure satisfactory patient survival and postoperative quality of life.

### Definitions and classifications of tumor margins

Given that the classification and definition of tumor margins continually evolve, with different studies adhering to various criteria (Table 1), this paper adopts precise definitions to circumvent potential ambiguity^28,29^.

**Table 1:** NCCN guideline’s interpretation of the margin of head and neck cancer.

Version	Classification and definition	Adequate excision
2010	Clear margin: ≥5 mm HSD Close margin: <5 mm HSD	Enough CSD (typically at least 2 cm) or clear frozen section margins
2011	Clear margin: ≥5 mm HSD Close margin: <5 mm HSD	Enough CSD to obtain clear frozen section and permanent margins (typically at least 1.5–2 cm)
2012–2013	Clear margin: ≥5 mm HSD Close margin: <5 mm HSD Positive margin: carcinoma in situ or invasive carcinoma at the margin of resection	Enough CSD to obtain clear frozen section and permanent margins (typically at least 1.5–2 cm)
2014–2019	Clear margin: ≥5 mm HSD Close margin: <5 mm HSD Positive margin: carcinoma in situ or invasive carcinoma at the margin of resection	Enough CSD to obtain clear frozen section and permanent margins (typically at least 1.5–2 cm) For glottic cancer: 1–2 mm In TLM: 1.5–2.0 mm
2020	Clear margin: ≥5 mm HSD Close margin: <5 mm HSD Positive margin: carcinoma in situ or invasive carcinoma at the margin of resection	Enough CSD to obtain clear frozen section and permanent margins (typically at least 1.0–1.5 cm) For glottic cancer: 1–2 mm In TLM: 1.5–2.0 mm
2021–2023	Clear margin: ≥5 mm HSD Close margin: <2–5 mm HSD, depending on the anatomic site involved Positive margin: carcinoma in situ or invasive carcinoma at the margin of resection	Enough CSD to obtain clear frozen section and permanent margins (typically at least 1.0–1.5 cm) For glottic cancer: 1–2 mm In transoral endoscopic and robotic approaches for oropharynx cancers: 1.5–2.0 mm

CSD, clinical surgical distance (the distance from gross tumor to the resected margin); HSD, histological surgical distance (the distance from the invasive tumor front to the resected margin); NCCN, National Comprehensive Cancer Network; TLM, transoral laser microsurgery.

The term ‘*tumor margin*’ delineates the tumor’s outer boundary, which can be further divided into clinical/gross margin, histological margin, and molecular margin. The ‘*clinical/gross margin*’ represents the macroscopic boundary ascertainable via clinical examination such as visual inspection and palpation. *Histological margin* demarcates the boundary between tumor cells (or morphologically abnormal cells) and normal cells. The *molecular margin*, on the other hand, refers to the boundary between genetically abnormal cells and their normal counterparts^30,31^.

**Figure 1:**
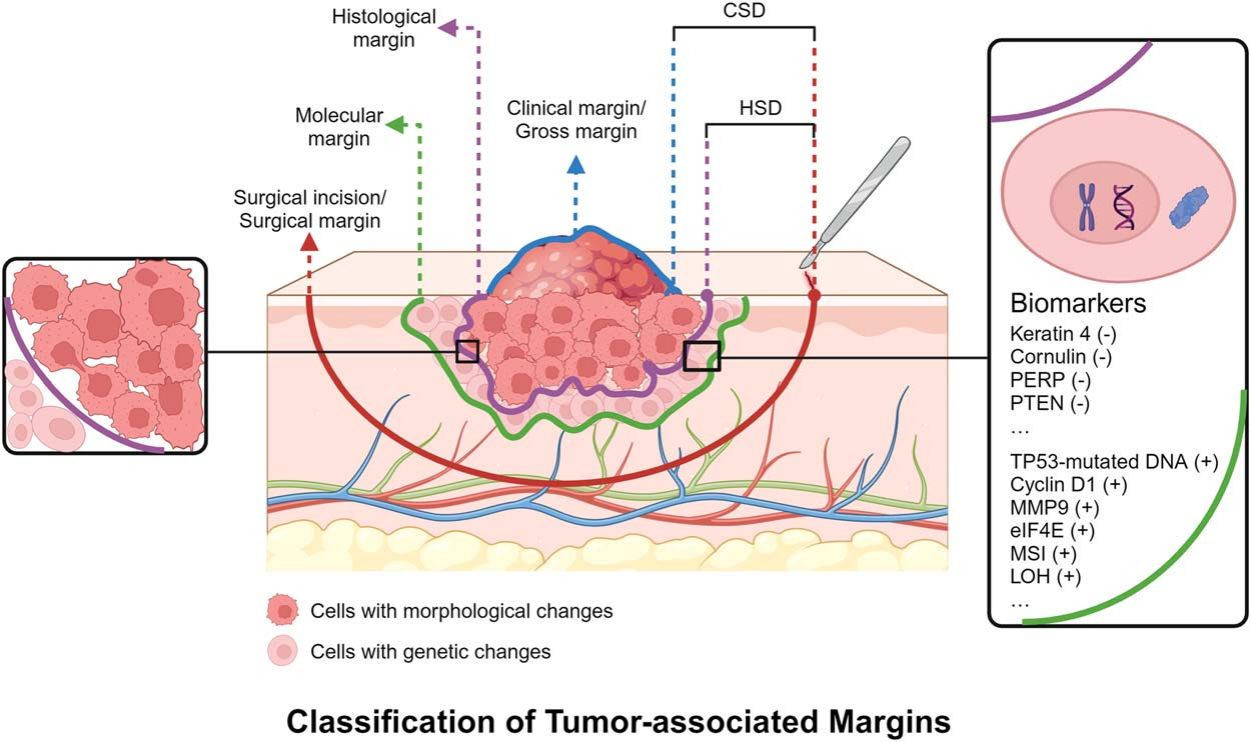
Schematic depiction of tumor-associated margins. This figure classifies tumor boundaries into three specific categories: the clinical or gross margin, visible macroscopically; the histological margin, and the molecular margin, both identifiable solely through microscopic analysis. Additionally, this review introduces two key measurements: the clinical surgical distance (CSD), which is the span between the clinical (gross) margin and the surgical margin, and the histological surgical distance (HSD), the interval between the histological margin and the surgical margin. Created with BioRender.com.

To avoid confusion with tumor margin, this review employs the term ‘*surgical margin* (SM)’ exclusively to denote the ‘surgical incision’, while ‘*surgical distance* (SD)’ refers to the gap between the surgical incision and the tumor margin^32^. Typically, the location of the SM is defined by the SD value. *Clinical SD* (CSD) indicates the span between the SM and the gross margin. Conversely, *histological SD* (HSD) signifies the distance between the SM and the tumor’s histological margin (Fig. 1).

The margin status is primarily categorized based on the SM’s distance from the tumor’s histological margin into *clear involved* (<1 mm HSD), *close* (1–5 mm HSD), and *involved clear* (>5 mm HSD)^29^. Additionally, based on the presence or absence of tumor cells (carcinoma in situ or invasive carcinoma) or dysplasia at the SM, margin status bifurcates into *positive* and *negative*, with the former generally indicative of an involved margin and the latter suggestive of a clear margin^30^. It is essential to note that the foundational understanding of the margins lacks universal consensus, with different studies adhering to various standards.

### Primary surgical margins

#### Surgical margin determination for diverse cancer types

The initial positive margin is significantly associated with inadequate marginal control and poor patient survival, thereby suggesting a potential surrogate for diffusely infiltrative cancer^33–35^. Notably, revisions to the positive margin have not led to significantly improved LC^36^. Consequently, the complete removal (R0 resection) of the tumor remains a fundamental surgical principle^37^. To achieve R0 resection with minimal loss of healthy tissue, investigators have undertaken extensive research on different types of HNSCC tumor specimens to determine the minimal safe SD.

*OSCC*: The oral cavity is the most frequent site of HNSCC, comprising the lip, buccal mucosa, hard palate, anterior tongue, floor of the mouth, and posterior molar triangle^38^. The long-standing belief has been that the oncologic target for surgery should exceed 5 mm HSD^39^. However, over the past decade, numerous retrospective studies have contested the 5 mm threshold. Consequently, the cut-off value for HSD varies significantly, primarily ranging from 1 to 7.6 mm for OSCC (Table 2).

**Table 2:** Cut-off value for histological surgical distance in oral squamous cell carcinoma.

	Publication date	Author	Patient number	Site	Evaluation items	Cut-off value for HSD
1	2008	Liao *et al*.^40^	827	OSCC	5-year LC	7 mm
2	2009	Nason *et al*.^41^	277	OSCC	5-year OS	3 mm
3	2010	Chiou *et al*.^42^	110	Buccal mucosa carcinoma	5-year LRR	3 mm
4	2010	Fan *et al*.^43^	302	OSCC	3-year LRC	4 mm
5	2012	Wong *et al*.^44^	192	OSCC, OPSCC	2-year DSS	1.6 mm
6	2016	Yamada *et al*.^45^	127	OSCC	5-year LR	5 mm
7	2017	Zanoni *et al*.^46^	381	TSCC	5-year LRFS	2.2 mm
8	2017	Tasche *et al*.^47^	432	OSCC	5-year LR	1 mm
9	2019	Mishra *et al*.^48^	602	Buccoalveolar SCC	5-year year LRFS	5.5 mm
10	2019	Kobayashi *et al*.^49^	284	OSCC	5-year LR	6 mm
11	2020	Jain *et al*.^50^	612	OSCC	1-year and 2-year DFS, OS, and LRRFS	1 mm (separate LRRFS and OS); 2 mm (separate DFS)
12	2020	Bajwa *et al*.^51^	669	OSCC	5-year year LRFS, DFS, and DSS	1 mm
13	2020	Brinkman *et al*.^52^	244	OSCC	LC, DSS, and OS	3 mm
14	2020	Daniell *et al*.^53^	258	TSCC	5-year LRC and OS	5 mm
15	2020	Singh *et al*.^54^	451	TSCC	LRRFS	7.6 mm
16	2021	Lin *et al*.^55^	15 654	OSCC	5-year CSS and OS	4 mm (separate CSS); 5 mm (separate OS)
17	2022	Woo *et al*.^56^	67	Maxillary OSCC	5-year OS and DFS	5 mm
18	2022	Fowler *et al*.^57^	412	OSCC	LRFS, recurrence-free survival, and OS	1 mm
19	2023	Otsuru *et al*.^58^	564	TSCC	LR	3.3 mm for horizontal distance; 3.1 mm for vertical distance

CSS, cancer-specific survival; DFS, disease-free survival; DSS, disease-specific survival; HSD, histological surgical distance; LC, local control; LR, local recurrence; LRC, local-regional control; LRFS, local recurrence-free survival; LRR, locoregional recurrence; LRRFS, locoregional recurrence-free survival; OS, overall survival; OSCC, oral cavity/oral squamous cell carcinoma; TSCC, tongue squamous cell carcinoma.

Despite these investigations, a consensus on the cut-off value for HSD in OSCC has yet to be reached. According to a 2023 meta-analysis incorporating seven studies, 4 mm emerges as a more significant cut-off value for distinguishing local recurrence (LR) rates compared to 5 mm. Nevertheless, any recommendations drawn from this analysis should be implemented cautiously^59^.

The SD for OSCC should be adaptable, subject to the particular circumstances. The first consideration should be the anatomical site and tissue characteristics. For instance, the prognosis for the upper gingiva is inferior compared to the lower gingiva in SCC patients, thus necessitating an appropriate extension of the former’s SD^60^. If OSCC has infiltrated the bone marrow, segmental mandibular resection could be justified, with the SD being 5 mm of soft tissue and 1 cm of hard tissue^61,62^. The second consideration is the three-dimensional (3D) expansion of the tumor. The posterior and deep SD may need enlargement due to the former’s association with survival and the latter’s link to overall recurrence^63,64^. The third factor is the distinction in microscopic tumor extension between early and advanced tumors, indicating that the SD can also be tailored according to the primary tumor’s size^65^. Lee *et al*. first demonstrated that an SD of 0.5 cm was adequate for early T-stage tongue cancer. However, for advanced T-stage tongue cancer, they recommended at least 0.95 cm of posterior SD and 0.80 cm of deep SD^64^. In contrast, Priya *et al*.^66^ previously reported that there was no statistically significant correlation between the T stage and the SD in oral cancer (*P*=0.193). A fourth consideration is the association between SD value and tumor aggressiveness^67^. Ota *et al*. pioneered the development of an objective classification system to guide the surgical approach, whether resection or preservation, based on the depth of tumor invasion in relation to the buccinator, its fascia, and the overlying fascia. According to their classification, the disease-specific survival rate and the LC rate of buccal SCC patients were 73.7% and 89.5% respectively^68^. Assuming a 10% LR rate postoperatively in HNSCC, an HSD of 3 mm for patients without perineural invasion and 5.5 mm for those with perineural invasion would be adequate^69,70^. Moreover, it is worth noting that the SD is also affected by the types of the worst pattern of invasion; for types 1–3, the ideal SD is 1.7 mm, whereas for types 4–5, it is 7.8 mm^71^. Finally, the intraoperative utilization of frozen section analysis (FSA) can facilitate more accurate SM determination^72^. In the context of early-stage lower lip SCC, several researchers have suggested that a 3 mm SD is sufficient when using FSA for evaluation. However, in the absence of FSA, the SD should be increased to at least 6 mm^73^.

The surgical management of tongue squamous cell carcinoma (TSCC) presents a noteworthy subject due to the organ’s intricate composition of muscles, nerves, blood vessels, and lymphatic networks, all of which potentially facilitate tumor proliferation. In 2011, Calabrese *et al*.^74^ introduced the principle of ‘compartmental surgery’ for advanced tongue cancer. This strategy involves resecting compartments – each hemi-tongue delineated by the lingual septum, stylohyoid ligament, and muscle, and mylohyoid muscle – containing the primary tumor. The objective of this approach is to ensure complete tumor removal while eliminating prospective spread routes^74,75^. In comparison to conventional surgery (≥1 cm CSD), compartmental surgery yielded better 5-year outcomes in terms of LC, an improvement of 16.8%; locoregional control (LRC), a 24.4% improvement; and overall survival (OS), a 27.3% improvement. This operative paradigm employed in compartmental surgery, centered on ‘quality margins’, may prove beneficial in the future surgical management of OSCC. This is due to anatomical barriers, such as the fascia of facial muscles, the fascia constituting the hyoid septum, the fascia surrounding the hyoid muscles, and the periosteum of the jaw, which could serve as defenses against tumor invasion, potentially equivalent to a 2–3 cm thickness of normal tissue. Otsuru *et al*. proposed a method for OSCC resection based on quality margins. In early-stage tongue cancer, distance determines the resection margin within a single component. However, disease progression may necessitate the use of quality margins. If the tumor spans multiple components like muscle and bone, the selection of either distance or quality margins is made component-wise^58^.

*OPSCC*: At present, studies on HSD in OPSCC are comparatively scarce. Given that the oropharyngeal wall’s thickness typically falls below 5 mm, it would be appropriate to decrease the SD in cases of OPSCC^76^. Lee *et al*. reported a significant association between an HSD of 1–5 mm and elevated 5-year disease-free survival (DFS) and OS rates, compared to HSD less than 1 mm. They demonstrated a survival rate of 65.1% versus 52% for DFS (*P*=0.034) and 84% versus 52.3% for OS (*P*=0.001)^77^. Hinni *et al*.^78^ further posited the feasibility of a minimum SD of 1.98 mm deep and 2.82 mm peripheral (*P*=0.0003).

*LSCC*: The degree of submucosal infiltration in LSCC correlates with the T stage, suggesting that a 5 mm HSD suffices for T1–2 LSCC, while T3–4 LSCC may necessitate an HSD of at least 10 mm^79^. Glottic SCC typically exhibits a narrower SD than other HNSCC. Alicandri-Ciufelli *et al*.^80^ advocated for a close margin of ≤1 mm in the vocal cord, and ≤5 mm in the larynx. Given that the vocal cords’ anatomical thickness is only 3–5 mm, and the sparse distribution of lymph nodes, preserving the vocal cords’ structural integrity is crucial for maintaining speech functions^81,82^. Currently, transoral laser microsurgery (TLM), as opposed to traditional surgery, is often favored for treating Tis-T2 Glottic SCC, primarily recommending a 1 mm SD^83,84^. However, assessing postoperative margins poses challenges due to the laser-induced thermal injury and cell carbonization, necessitating the surgeon’s comprehensive understanding of TLM, including factors such as the laser spot size, power, and depth of artifactual thermal burns^85,86^.

*HPSCC*: In HPSCC, the microscopic extensions significantly varied by T status, with submucosal microscopic extensions in T1–2 tumors being <5 mm, while those of T3–4 tumors ranged between 1.5 and 2.0 cm^87^. Furthermore, the frequencies of submucosal spread in HPSCC, from highest to lowest, occurred in the medial (37%), inferior (28%), lateral (26%), and superior (16%) directions. Correspondingly, the limit of submucosal tumor extension was 25, 20, 20, and 10 mm^88^. Hence, these data should guide the adjustment of CSD in HPSCC^89,90^.

*NPC*: Liu *et al*. provided evidence supporting the feasibility of performing endoscopic nasopharyngectomy for select patients with localized stage I NPC. The surgical procedure was based on the planned surgical tumor volume, defined as the gross tumor volume in combination with an additional 0.5–1.0 cm peripheral mucosa SD and a 2–3 mm basal SD on the surface skull base. According to the magnetic resonance imaging (MRI) and endoscopic results, no recurrence or metastasis developed during 3-year follow-up^91^. Overall, while radiotherapy remains the primary treatment for non-metastatic NPC, the radical resection of primary NPC warrants further investigation^92^.

Generally, per the National Comprehensive Cancer Network (NCCN) Guidelines Version 2.2023, a clear margin is >5 mm for HNSCC and 1–2 mm for glottis cancer^20^. To ensure thorough tumor removal, the CSD should exceed the recommended HSD, primarily because microscopic tumor foci might be present beyond palpable and visual margins, and tissue undergoes hard-to-quantify shrinkage during the transition from living tissue to specimen^21,70,93,94^. To achieve an HSD of 5 mm, a CSD of 1.0–1.5 cm is typically necessary to be excised^20^.

#### Limitations and challenges

Although a wealth of studies exists pertaining to the SMs of HNSCC, crafting individualized SM remains a challenge for surgeons due to the inherent complexities of real-world clinical scenarios.

**Figure 2:**
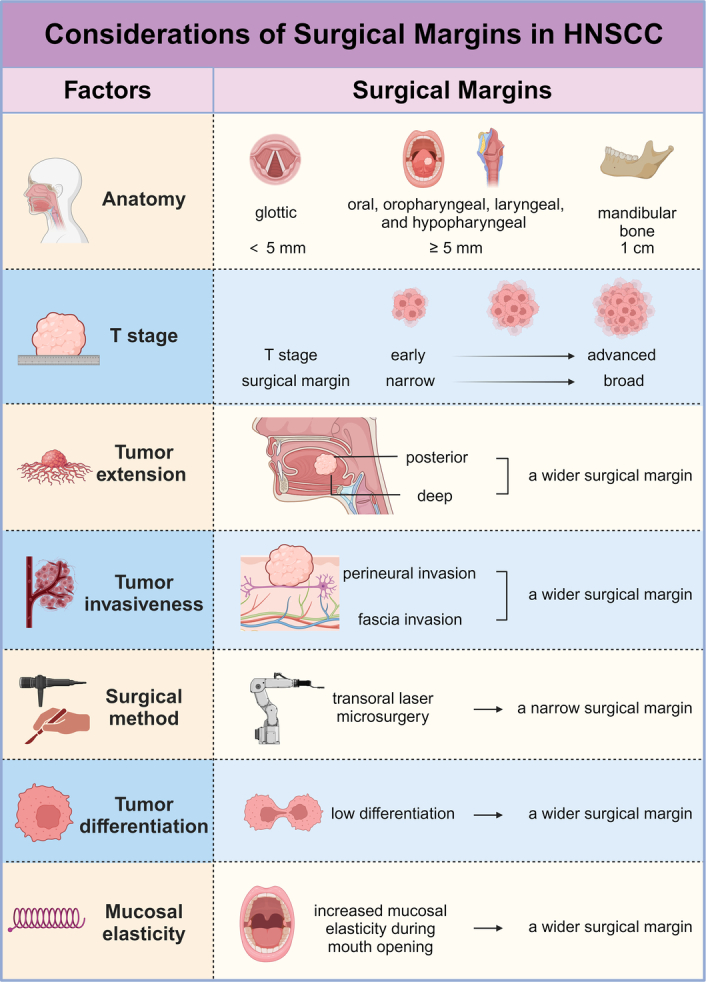
Considerations of surgical margins in head and neck squamous cell carcinoma (HNSCC). The determination of surgical margins in HNSCC necessitates comprehensive considerations of numerous factors. These include the anatomical location and characteristics of the tumor, T stage, the three-dimensional extent of the tumor, tumor invasiveness, the chosen surgical approach, the degree of tumor differentiation, and mucosal elasticity. Created with BioRender.com.

On the one hand, typical values for HSD are often distilled from retrospective studies, yet a robust SM strategy should be underpinned by evidence-based medicine. However, as of now, there are scarce evidence-based studies concerning HSD in HNSCC, and those that do exist present limitations, such as the absence of prospective studies and a dearth of subgroup analyses with regard to covariates^26,59^. Although more studies are necessary to provide reliable SMs, the ethical concerns related to conducting prospective trials may prove a significant hurdle^48^. SM planning should take into account multiple factors, including anatomical location and characteristics, three-dimensional tumor extension, T stage, tumor invasiveness, and the surgical approach^95^. For cases of invasive SCC, poorly differentiated SCC, and cases localized to the ear or lip, a wider SD is necessary^96^. Considering that mucosal elasticity increases during maximum mouth opening in patients with buccal SCC, thereby affecting tumor size and adjacent mucosa, the estimated SD should be amplified when performing transoral resection of buccal SCC^97^ (Fig. 2). Overall, more comprehensive evidence-based studies, inclusive of subgroup analyses, are needed to provide more reliable SM guidelines.

On the other hand, while our comprehension of HSD cut-off values is continually advancing, translating this understanding into CSD is often confounded by variations in tissue shrinkage, contingent upon factors such as anatomical sites, stage, resection technique, and tissue processing methodology^98^. Currently, some studies have endeavored to quantify tissue sample shrinkage rates^99^, and various strategies have been proposed to mitigate shrinkage-related issues, such as the basal cell counting method, as well as other intraoperative analytical techniques, including FSA, microendoscopy, optical coherence tomography, and elastic scattering spectroscopy^100–102^. Moreover, discrepancies frequently emerge between the surgeon’s evaluation of the resection status and the pathological examination of the resected specimen based on intraoperative FSA.

Crucially, we must not focus solely on margin status, as other critical risk factors, including depth of invasion and extranodal extension, may provide a more accurate prognosis^63,103,104^.

### Revised surgical margins

Undoubtedly, the mastery of appropriate SMs for primary resection is of paramount importance^35^. Yet, it is not infrequent that intraoperative pathological examinations reveal close or involved margins. In these cases, re-resection of the primary tumor bed is advocated to achieve R0 status, rendering the extent of the revised margin equally significant^105^.

A survey by Bulbul *et al*.^106^ directed at 185 members of the American Head and Neck Society, it was found that 96.8% of respondents utilized FSA to inform additional resection of OSCC. As per Brandwein-Gensler *et al*.^107^, the revisionary treatment approach for OSCC patients involved resecting further tissue if the intraoperative FSA exhibited a ≤5 mm HSD, with the examination and revisionary steps repeated until satisfactory resection. Sifrer *et al*. pursued a similar approach; if FSA demonstrated a negative margin, they concluded the operation, which could be considered the initial R0 resection. If, however, FSA indicated a positive margin, they executed a secondary 5 mm SD^108^.

Concerning perceptions of negative margins achieved via revision surgery, an early questionnaire indicated that 90% of respondents equated a revised negative margin with an initial negative margin^109^. Moreover, a retrospective study in 2010 demonstrated equivalent survival rates between the R1–R0 group (clear margins achieved following repeated resection) and the R0 group (immediately clear margins) (2-year OS: 90% vs. 91%; 5-year OS: 72% vs. 76%)^110^. However, a recent meta-analysis reported that the local recurrence-free survival (LRFS) of the R1–R0 group was inferior to the initial R0 group [hazard ratio (HR)=2.897, *P*<0.001]^36^. The R1–R0 group could not match the LC and distant control rates of the initial R0 group (66.2% vs. 82.8%, *P*=0.045; 86.3% vs. 100.0%, *P*=0.021), despite similar regional control rates (85.1% vs. 90.2%, *P*=0.130)^111^. Thus, while additional resection based on FSA may improve patient outcomes to some degree, it remains consistently inferior to the initial R0 resection^36,112^.

Margin revisions, guided by intraoperative FSA, often prove insufficient, likely due to the challenges of re-identifying residual tumors and estimating the volume for supplementary resection^113,114^. A prospective study revealed that the average error in repositioning a site for mucosal and deep margins was 9 mm and 12 mm, respectively^115^. Effective methods for repositioning residual tumors include marking margin points with sutures or employing paired tagging^115,116^. An innovative method that uses targeted fluorescence imaging in conjunction with FSA swiftly and effectively identifies the ‘sentinel margin’ of specimens, thus offering potential guidance for additional resection in the tumor bed^117^.

It is crucial to note that with TLM, residual tumors are often undetectable until second-look microlaryngoscopy, irrespective of the primary resection margin status. This is due to the obscuring effects of laser-induced tissue damage^86,118^. Therefore, ample time should be allocated between reoperation and initial CO_2_ laser treatment to facilitate adequate tissue healing^119^. Most literature suggests that if the margin is positive, a second-look TLM is unequivocally necessary, and if the margin is close or non-valuable, close monitoring or a second-look TLM is recommended^120^. Given that inadequately resected sites can be treated via secondary transoral approaches, revisionary treatment after TLM was feasible, and even TLM had high efficacy in revision specimens with residual lesions^121^.

In general, be it intraoperative margin revision or a second TLM, the problem of tumor relocating persists. Since a revised negative margin is not equivalent to an initial negative margin, postoperative adjuvant radiotherapy or CRT can provide a more reliable means of improving disease control and OS^22,122^. The challenge of executing precise SM revision remains^70,123^. Real-time intraoperative assessment technology may present an effective solution^124^. Moreover, the necessity of adjuvant therapy for patients undergoing revisionary surgery is another topic meriting further exploration^125^.

### Surgical margins after neoadjuvant therapy

Neoadjuvant therapy is a preoperative systemic treatment that can reduce the tumor stage and lessen the risk of distant metastasis^126,127^. While it is theoretically plausible that the successful response of certain tumors to neoadjuvant therapy might allow for a reduction in CSD, this notion is complicated by several factors. The unpredictable nature of tumor shrinkage due to heterogeneous responses to neoadjuvant therapy, along with the adhesion, fibrosis, and inflammatory reactions induced by such therapy, can significantly obfuscate the boundaries of residual tumors. Consequently, this presents a considerable challenge in executing precise surgical interventions post-neoadjuvant therapy in HNSCC^126,128^. Currently, definitive guidelines pertaining to the extent of surgery (be it according to the original tumor margins or post-treatment tumor margins) following neoadjuvant chemotherapy (NACT) or neoadjuvant immunotherapy (NAIT) are yet to be established^129^.

#### The role of neoadjuvant chemotherapy in reshaping surgical margins

NACT, also referred to as induction chemotherapy, serves as a preliminary treatment prior to the execution of radical surgery or CRT^130^. Due to its potential for downstaging tumors, a combination of NACT and surgery has become a prevalent therapeutic approach for resectable LAHNSCC. It is also considered an alternative treatment pathway for cases deemed inoperable^131,132^.

NACT has been demonstrated to potentially convert some unresectable tumors into resectable ones, enabling certain patients to preserve vital organs such as the larynx and eyes without compromising survival rates^133–136^. Regarding the extent of surgery after NACT, given the limitations in assessing tumor response and the apprehensions surrounding non-concentric tumor contraction, most surgeons tend to favor a surgical approach based on primary tumor characteristics^109,137–139^. Historical practices dating back to the 1970s saw the use of tattoo materials such as India ink to mark the original lesions and guide surgeons toward performing radical surgery after neoadjuvant treatment^140,141^. To this day, a CSD of over 10–15 mm is consistently advocated for, yielding satisfactory oncological and functional outcomes^142^. Interestingly, a study published in Chinese reported that the detection rate of cancer cells was significantly lower in the post-NACT radical surgery group for macroscopic hypopharyngeal cancer (T2–3) compared to the upfront radical surgery group, suggesting a feasible reduction in the CSD after NACT (*P*<0.05)^143^.

With the continuous pursuit of aesthetic and functional outcomes combined with the considerable advances in chemotherapy drugs and treatment techniques, research into the feasibility and safety of limited surgery after NACT has progressively surfaced over the past decade. The notion that NACT plus reduced CSD may enable future organ preservation was first proposed in 2008^144,145^. Given the improved efficacy of contemporary medication regimens, such as the combination of paclitaxel, cisplatin, and 5 fluorouracil, the SM after NACT warrants further discussion^146^. Lee *et al*.^147^ were the pioneers in constructing a murine HNSCC model, finding no significant difference in recurrence and survival rates between the post-NACT limited surgery group (surgery was dictated by residual tumor) and the post-NACT radical surgery group, indicating the potential safety and effectiveness of limited surgery after NACT. A retrospective analysis by Schmaltz *et al*.^132^ demonstrated that surgery performed according to residual tumor could achieve R0 resection in 85.7% of patients. Regarding the positive margin rate, the margin status of post-NACT limited surgery was comparable to that of upfront surgery (*P*=0.212, *P*=0.519)^148,149^. Furthermore, a phase II randomized trial by Chaukar *et al*.^150^ indicated that the intervention arm (NACT combined with limited surgery) displayed similar OS and DFS rates as the standard arm (upfront surgery combined with adjuvant treatment), with an acceptable toxicity profile. Lee *et al*. demonstrated that there was merely a single instance of a positive margin and one case of LR, corroborating the safety of the surgical principle that mandates a CSD from the residual tongue tumor to be in excess of 1 cm. However, this clinical trial’s validity was constrained by a small sample size and the exclusion of tumors involving the gingiva or mandible, which may exhibit non-concentric shrinkage post-NACT^151^.

In conclusion, while NACT shows promise in reshaping the surgical approach for HNSCC patients, patient selection remains a crucial factor. For instance, in breast cancer, the 2017 St. Gallen International Expert Consensus Conference proposed that the extent of the residual tumor should guide the scope of breast surgery, with R0 removal always being the surgical standard. In instances of multifocal or scattered regression after NACT, a more generous CSD is recommended^152^. In HNSCC, a retrospective study by Kiong *et al*. identified tumor multifocal regression after NACT as a significant predictor of worse 3-year LRC compared to unifocal tumor and no viable tumor group (52% vs. 69% vs. 82%, *P*=0.015), although it did not significantly affect 3-year OS (60% vs. 65% vs. 71%, *P*=0.199). Their multivariate analysis also identified both multifocal regression (HR=10.43, *P*=0.031) and extranodal extension (HR=4.4, *P*=0.004) as significant independent predictors of LRC. Major pathological response (1–10% viable tumor) was associated with significantly better 3-year OS and LRC^126^. Therefore, it could be hypothesized that similar to breast cancer, a new SM defined according to the residual tumor should be considered in cases of HNSCC where concentric contractions or major pathological responses have been achieved. However, further evidence is required to substantiate this hypothesis. Given that NACT often blurs the lesions delineated by preoperative examinations, there is a pressing need for more accurate tumor margin assessment methods to attain concordance between imaging and histopathology^153,154^.

#### The role of neoadjuvant immunotherapy in reshaping surgical margins

The preoperative application of immune checkpoint inhibitors signifies an emerging therapeutic strategy for HNSCC. Often combined with radiotherapy, chemotherapy, and targeted therapy, this approach, collectively referred to as NAIT, functions differently than NACT, but can similarly reduce tumor size preoperatively and even render previously unresectable tumors operable^129,155^.

Currently, surgical procedures post-NAIT primarily follow a course of radical resection guided by the original tumor extent^156^. However, compelling evidence, including clinical-to-pathological downstaging rates and complete pathological response rates of up to 100% (ChiCTR1900025303) and 90% (NCT03247712), respectively, suggests promising prospects for reducing CSD after NAIT^157,158^. Preliminary evidence supporting reduced CSD after NAIT was provided by Leidner *et al*.^158^, who conducted surgeries based on the residual tumors, achieving 1-year OS and 1-year DFS rates of 100% and 95%, respectively. For certain elderly or frail patients with LAHNSCC, considering surgery avoidance might be feasible if they nearly or completely achieve a complete response to NAIT^159^. Generally, research on SMs post-NAIT is lacking; however, there is optimism about the potential for achieving narrower CSD to further enhance patients’ quality of life. For instance, neoadjuvant CRT combined with immunotherapy has broadened opportunities for selected patients with low rectal cancer to adopt a ‘Watch and Wait’ approach or undergo sphincter-preserving surgery^160^. Additionally, combining nivolumab with NACT has improved access to minimally invasive surgery for patients with non-small cell lung cancer^161^.

To the best of our knowledge, only two clinical trials have investigated SMs after neoadjuvant treatment in HNSCC. One anticipated to run from 2022 to 2026 (NCT05872880) involves patients with locally advanced OSCC who exhibit ≥50% tumor reduction after NACT and can choose between radical surgery (an SD of 1–1.5 cm outside the primary tumor) or modified radical surgery (an SD of 1–1.5 cm outside the residual tumor) according to their preference. Another trial, expected to run from 2022 to 2027, proposes that patients with HPV-negative LAHNSCC showing significant tumor shrinkage (≥50%) after a combination of NACT and immunotherapy would achieve reduced SD, with the 2-year DFS being the primary outcome measure (NCT05459415).

### Surgical margins of recurrent head and neck squamous cell carcinoma

HNSCC exhibits a proclivity for recurrence, with roughly half of the patients diagnosed with locally advanced disease undergoing relapse within a 2-year period post-treatment^162^. If patients remain recurrence-free for 5 years following treatment, they are generally considered disease-free^163^. LR mechanisms are multifactorial; minimal residual cancer and the emergence of a second primary tumor are the most frequently observed oncological explanations for LR^164^. Other plausible mechanisms encompass field cancerization, tumor implantation, and a comprehensive failure of the immune system^165,166^. Salvage surgery (SS) is the principal treatment option for resectable recurrent HNSCC, although less than 20% of patients with recurrent disease qualify for this procedure^167,168^.

Past surgical resection and reconstruction, alongside the impact of RT and chemotherapy, can lead to anatomical disruptions and affect the blood supply to tissues. These treatments can also induce specific mutations and changes in the biological behavior of tumor cells^169^. Consequently, it becomes challenging to accurately delineate the histological margins of recurrent HNSCC, making the assessment of clear margins particularly difficult^170^. Post-SS, it is essential to identify patients at high risk of secondary recurrence and those suitable for adjuvant therapy. Notably, there is a significant correlation between margin status and patient survival, as well as the risk of a second recurrence after SS^171^.

Given these considerations, SM evaluation during SS emerges as a pressing issue, and achieving clear margins remains a pivotal surgical objective^109,172^. In the context of locally recurrent NPC, the resection process should eradicate the tumor and secure an adequate mucosal margin, including the cartilaginous part of the Eustachian tube. Moreover, paranasopharyngeal tissues should be excised as indicated^173^.

## Assessment of head and neck squamous cell carcinoma margins

### Histology

**Figure 3:**
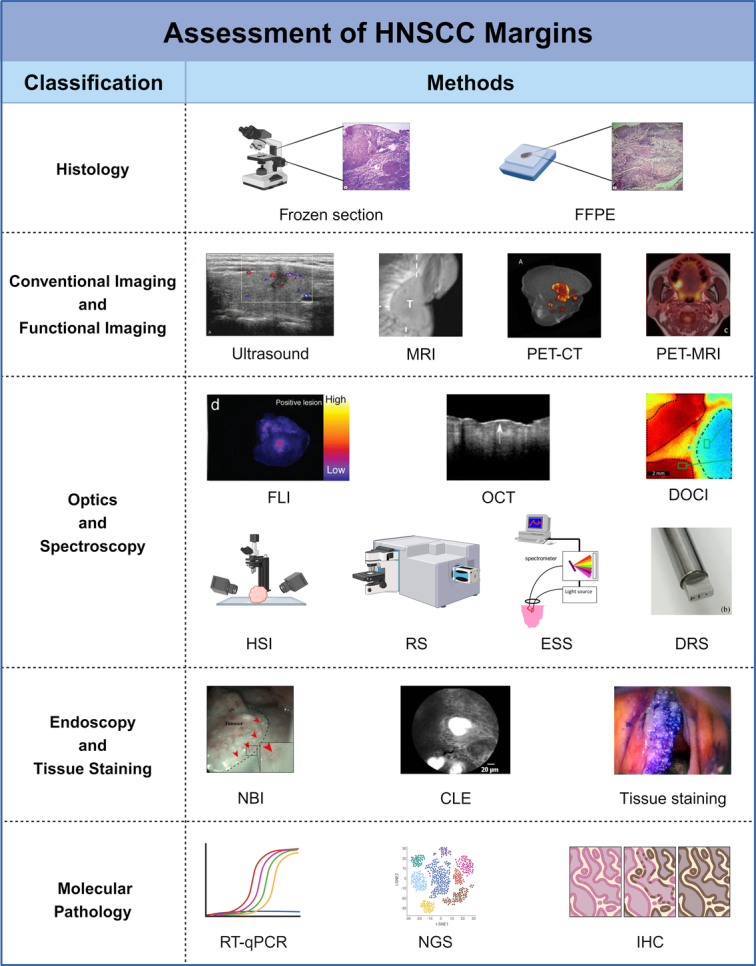
Techniques for assessing margins in head and neck squamous cell carcinoma (HNSCC). Advances in technology have led to a diverse range of methods for delineating HNSCC margins. These techniques are broadly categorized into histology; conventional and functional imaging; optics and spectroscopy; endoscopy and tissue staining; and molecular pathology. CLE, confocal laser endomicroscopy; DOCI, dynamic optical contrast imaging; DRS, diffuse reflectance spectroscopy; ESS, elastic scattering spectroscopy; FFPE, formalin-fixed paraffin-embedded; FLI, fluorescence imaging; HSI, hyperspectral imaging; IHC, immunohistochemistry; MRI, magnetic resonance imaging; NBI, narrow-band imaging; NGS, next-generation sequencing technology; OCT, optical coherence tomography; PET-CT, positron emission tomography – computed tomography; PET-MRI, positron emission tomography – magnetic resonance imaging; RS, Raman spectroscopy; RT-qPCR, real-time quantitative polymerase chain reaction. Figure adapted from^174–186^ under Creative Commons Attribution License (CC BY). Created with BioRender.com.

As stipulated by the NCCN Guidelines Version 2.2023, the primary objective of oncologic surgery is the comprehensive resection of the tumor, ensuring histologically confirmed tumor-free margins. This assessment is predominantly carried out using fresh frozen sections or formalin-fixed, paraffin-embedded (FFPE) sections (Fig. 3)^20^.

Currently, FSA is the most widely utilized method for intraoperative margin assessment. A significant portion of the medical community believes that performing FSA on resected tissue samples, rather than on the tumor bed, provides a more accurate evaluation of margin status^187^. A cross-sectional study investigating 432 tumor margins found FSA to have a sensitivity, specificity, positive predictive value (PPV), negative predictive value (NPV), and accuracy of 50%, 99.8%, 75%, 99.3%, and 99%, respectively, in contrast with the permanent section^188^. However, FSA is not without limitations. It is subject to sampling errors during analysis and, while its specificity is high, exhibits low sensitivity in predicting margin status^189^. Moreover, the process is both time- and labor-intensive^190^. Other potential drawbacks include the need for clear communication between the surgeon and the pathologist, and challenges in locating intraoperative revised SMs^189^.

Moving forward, the standardization of FSA is required, including improvements in data collection and enhanced surgeon–pathologist communication, to augment the accuracy of margin assessment^36^. Alternatively, the adoption of improved imaging technologies could also contribute to this endeavor^191^.

### Conventional imaging and functional imaging

Ultrasound serves as a practical intraoperative tool for assessing deep margins and determining the depth of infiltration, rendering it the gold standard for preoperative evaluation of OCSCC^192,193^. Compared to conventional surgery, ultrasound-guided surgery significantly enhances margin status and reduces the need for adjuvant therapy (free margin status: 55% vs. 16%, *P*<0.001; positive margin: 5% vs. 15%, respectively, *P*<0.001)^194^.

MRI demonstrates commendable accuracy. When compared with intraoperative tumor profile images, the false-positive and false-negative MRI values for TSCC margin assessment were 1.52±0.87 mm [95% limit of agreement (LoA) 1.36–1.64], and 0.35±0.20 mm. Similarly, when compared with postoperative pathological sections, these values were 1.95±1.39 mm (95% LoA 1.70–2.14) and 0.44±0.49 mm, respectively^195^. Giannitto *et al*. pioneered the use of a 3D-printed tongue model for specimen orientation, subsequently employing ex-vivo MRI for margin assessment. This novel method has been proven to enhance the accuracy of margin status evaluation^186^.

Functional imaging (FI) technology, which encapsulates positron emission tomography (PET) and functional MRI technology, enables the capture of the tumor’s metabolic information^196,197^. PET-computed tomography (PET-CT), a molecular and FI modality, shows promise in assessing both superficial and deep margins of head and neck malignancies^176,198^. However, the exclusive use of PET-CT imaging is inadequate for distinguishing between inflammatory and malignant tissue^199,200^. To improve this differentiation, a combination of FI and cone-beam CT has been introduced to evaluate medication-related osteonecrosis of the jaw, demonstrating promising results by not only providing accurate anatomical images but also illustrating the dynamics of bone viability, bone remodeling, and inflammatory activity^201^. Gadolinium-enhanced MRI sequences contribute to better determination of tumor margins without increased diagnostic accuracy^202^. PET-MRI surpasses MRI and PET-CT in detecting bone and soft tissue invasion and is a promising tool for delineating SMs in HNSCC, though it falls short in guiding biopsy by delineating genetic heterogeneity, according to limited data^197,203^. Recent developments in FI techniques provide multi-dimensional and computer-assisted navigation for surgeons, with a new 3D-navigation system based on PET-CT image fusion showing great potential in evaluating SMs and improving LC of advanced HNC^204,205^.

Fractal dimension (FD), which quantifies a pattern’s complexity by comparing how detail changes with the scale at which it is measured, presents a promising method to objectively distinguish dysplasia and carcinoma from normal mucosa^206^. FD, a quantitative result of fractal analysis applied to radiological perfusion imaging, represents chaos in the perfusion pattern and can be related to the structure of the underlying vascular tree^207–209^. For HNSCC, FD has proven useful in the quantitative analysis of the epithelial–connective tissue interface and prediction^210–213^. Klatt *et al*.^214^ reported that FD, based on time-resolved autofluorescence spectra, achieved 86% specificity and 100% sensitivity in distinguishing carcinoma from healthy oral tissues. Moreover, a 2017 study established FD as a novel tool for differentiating normal tissue from dysplastic and neoplastic tissue, suggesting that fractal geometry holds promise in studying both physiological and pathological changes in the oral mucosa^215^.

### Optics and spectroscopy

Fluorescence imaging (FLI), a prime example of optical imaging, exhibits high sensitivity and spatial resolution, although it is constrained by a limited penetration depth (several millimeters)^188^. Predominantly, it serves in early cancer detection and aids in SM evaluation during surgical procedures^216–218^. In delineating the SMs of HNSCC, an innovative fluorescent-labeled antibody technique, panitumumab-IRDye800, demonstrated 95% sensitivity and 89% specificity for oral cancer margin detection^219^. Additionally, an ex-vivo FLI technique in combination with ONM-100, a promising pH-dependent imaging agent, can accurately discriminate non-tumor from tumor tissues and identify positive margins with 100% sensitivity^178^. Moreover, owing to its capacity to decrease abnormal rates at SMs from 6.25% to 0.78% (*P*<0.05), indocyanine green-based near-infrared fluorescence molecular imaging has proven to be an effective intraoperative tool for residual tumor detection^220^. Compared to contrast agent-assisted FLI, label-free FLI offers rapidity and convenience. Marsden *et al*.’s study^221^ demonstrated that integrating label-free fluorescence lifetime imaging with machine learning (ML) facilitates fast, reliable intraoperative margin assessment by capturing radiance differences between healthy and cancerous tissues. Optical coherence tomography also emerges as a promising non-invasive tool for real-time intraoperative margin assessment, permitting images to be captured up to 2 mm from the mucosal surface^222,223^. Furthermore, dynamic optical contrast imaging, a novel and real-time wide-field technique, distinguishes tumors from adjacent healthy tissue based on fluorescence decay information from the UV-VIS spectral bands^224^.

Currently, spectral techniques for assessing HNSCC margins primarily include hyperspectral imaging (HSI), Raman spectroscopy (RS), elastic scattering spectroscopy (ESS), and diffuse reflectance spectroscopy. Hyperspectral imaging (HSI), a noncontact, label-free, and reflectance-based imaging modality, can accurately differentiate the tumor margin in ex-vivo specimens within minutes, exhibiting an area under the curve (AUC) of 0.85–0.95^190^. RS, an intraoperative margin assessment technique implemented in real-time, is based on water concentration discrimination, showing a high sensitivity and specificity of 99% and 92%, respectively^225^. ESS is a fast, real-time method that distinguishes normal from abnormal tissue, demonstrating a sensitivity of 84–100% and a specificity of 71–89%^181^. Additionally, the handheld diffuse reflectance spectroscopy probe accurately delineates the oral cancer margin and guides precise resection, delivering a sensitivity, specificity, and accuracy of over 80%^182^.

### Endoscopy and tissue staining

Endoscopy serves as a pivotal tool in distinguishing between normal and abnormal tissue, augmenting the arsenal of imaging techniques available for diagnostic purposes. One such non-invasive endoscopic approach is narrow-band imaging (NBI). The sensitivity, specificity, PPV, and NPV of NBI in diagnosing dysplasia and identifying positive margins were found to be 100% and 88.9%, respectively, with its PPV and NPV standing at 100% and 87.5%, respectively^226^. Despite its efficacious results, the limitation of NBI lies in its inability to detect submucosal extensions beyond a penetration depth of 240 μm^183^.

A budding technology, confocal laser endomicroscopy (CLE), offers real-time histological visualization of living tissues, thus adding to its potentiality. For laryngeal tumors, the accuracy, sensitivity, specificity, PPV, and NPV of CLE were reported as 80.1%, 72.3%, 87.9%, 85.7%, and 76.1%, respectively^227^. In the context of OPSCC, CLE yielded results of 86%, 90%, 79%, 88%, and 82% in the aforementioned categories, respectively^184^.

Additionally, in-vivo tissue staining techniques such as Lugol’s iodine and toluidine blue staining serve as safe, economical, and user-friendly adjunct examination methods. They have a well-established history in diagnosing oral precancerous lesions and tumors, and in defining dysplastic epithelium or tumor boundaries. Presently, these techniques are commonly employed to delineate the extent of glottic cancer to guide TLM^185,228^.

### Molecular pathology

In the surgical treatment of HNSCC, maintaining a clear margin remains a fundamental principle. Traditional histopathological and radiological examinations, however, present limitations in detecting minimal residual cancer and preneoplastic cells, thus heightening the risk of patient relapse^164,229,230^. Molecular diagnostics, with its advancements, has begun to play an increasingly vital role in discerning tumor boundaries, principally due to its capacity to detect early genetic alterations even at the margins of phenotypically normal tissues^231^.

Contemporary research on the molecular margin of HNSCC is progressively expanding, yielding promising biomarkers that are detailed in Table 3 ^79,232–237^. This burgeoning field of study underscores the potential of molecular analysis in enhancing the precision of surgical interventions for HNSCC.

**Table 3:** Promising biomarkers of molecular margin for head and neck squamous cell carcinoma.

	Biomarkers	Cancer types	Detection method	Correlation (with poor outcomes)
1	TP53-mutated DNA	HNSCC	PCR	Positive
2	MSI	HNSCC	PCR	Positive
3	eIF4E	HNSCC	IHC	Positive
4	Cyclin D1	HNSCC	IHC	Positive
5	Osteopontin	HNSCC	IHC	Positive
6	LOH	HNSCC	IHC	Positive
7	Keratin 4	HNSCC	IHC	Negative
8	Cornulin	HNSCC	IHC	Negative
9	PERP	HNSCC	IHC	Negative
10	MMP9	HNSCC	qRT-PCR	Positive
11	PTHLH	HNSCC	qRT-PCR	Positive
12	EGFR	HNSCC	qRT-PCR	Positive
13	CDKN2A	HNSCC	QMSP	Positive
14	PAX5 gene promoter methylation	HNSCC	QMSP	Positive
15	Survivin	Laryngeal carcinoma	IHC	Positive
16	CD44v6	Laryngeal carcinoma	IHC	Positive
17	PCNA	Laryngeal carcinoma	IHC	Positive
18	Bcl-2	Laryngeal carcinoma	IHC	Positive
19	PTEN	Laryngeal carcinoma	IHC	Negative
20	P53	Laryngeal carcinoma	IHC	Positive
21	P27	Laryngeal carcinoma	IHC	Negative
22	CNA in chromosomes 1 and 7	OSCC	I-FISH	Positive
23	The methylated gene combination of EDNRB and HOXA9	OSCC	QMSP	Positive
24	DAPK	OSCC	Multiplex nested methylation-specific PCR	Positive
25	Axin2	OSCC	IHC	Positive
26	Snail	OSCC	IHC	Positive

Bcl-2, B cell lymphoma 2; CDKN2A, promoter hypermethylation of the p16 gene; CNA, copy number alteration; DAPK, death-associated protein kinase; EDNRB, endothelin receptor type B; EGFR, epidermal growth factor receptor; eIF4E, eukaryotic translocation initiation factor 4E; HNSCC, head and neck squamous cell carcinoma; HOXA9, homeobox protein A9; I-FISH, interphase fluorescence in situ hybridization; IHC, immunohistochemistry; LOH, loss of heterozygosity; MMP9, metallopreoteinase-9; MSI, microsatellite instability; OSCC, oral squamous cell carcinoma; PCNA, proliferating cell nuclear antigen; PCR, polymerase chain reaction; PERP, p53 apoptosis effector related to PMP22; PTEN, phosphatase and tensin homolog; PTHLH, parathyroid hormone-like hormone; QMSP, quantitative methylation-specific polymerase chain reaction; qRT-PCR, quantitative reverse transcription polymerase chain reaction.

### The confluence of artificial intelligence and assessment techniques

**Figure 4:**
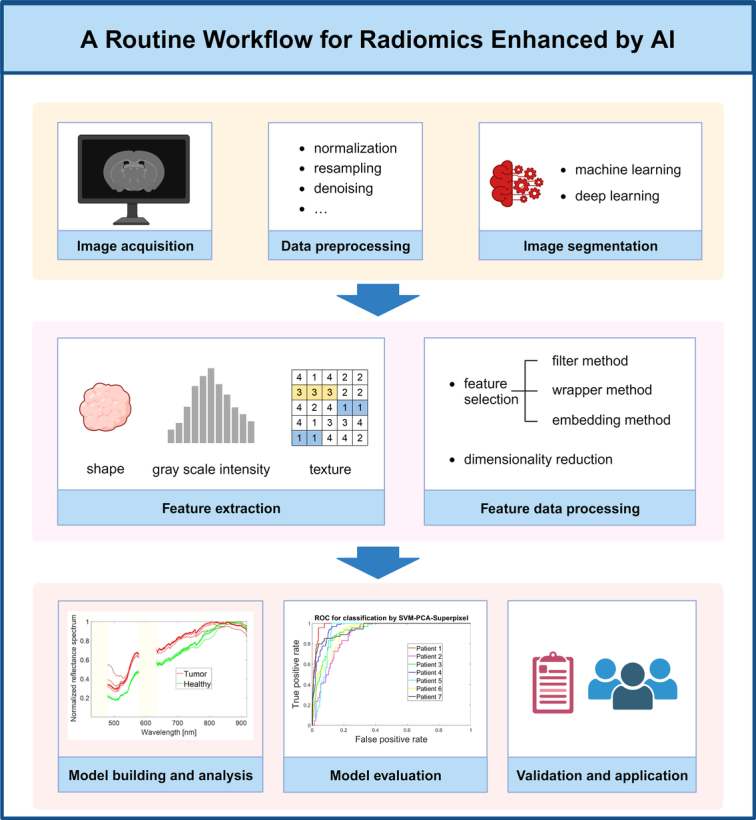
A routine workflow for radiomics enhanced by artificial intelligence (AI). In the field of radiomics, AI is pivotal, particularly in analyses involving machine learning. The typical workflow comprises several stages: image acquisition, data preprocessing, image segmentation, feature extraction, feature data processing, model building and analysis, model evaluation, and finally, the validation and application of these models. Figure adapted from^240^ under the Creative Commons Attribution License (CC BY). Created with BioRender.com.

In light of the considerable volume of imaging data now available and the enhanced computational power of contemporary computer processors, the field of artificial intelligence (AI) presents significant potential for image recognition and analysis^238^. Originally conceived in 1956, AI represents an area of research where machines emulate human cognitive functions^238,239^. Specifically, ML and deep learning (DL), subdivisions of AI, can be employed in the initial acquisition and processing of images, expedited assessment and enhancement of image quality, and the autonomous detection, interpretation, and reporting of findings^238^. ML employs statistical methods to discern concealed patterns within a dataset, thereby achieving AI. Meanwhile, DL, as a subset of ML, leverages the capabilities of neural networks^239^. In recent years, AI has played an important role in radiomics analysis (Fig. 4).

With respect to the segmentation of head and neck lesions, the synergy of AI with assessment methodologies can not only address the limitations of FSA, such as time consumption and subjectivity, but can also more rapidly distinguish and quantify lesion features, and improve image quality (for instance, by reducing metal artifact, cross-talk noise, and magnetic field inhomogeneity)^238^. Currently, the amalgamation of AI and optical and spectroscopic imaging techniques has yielded encouraging advancements in the evaluation of HNSCC. As per Manni *et al*.^240^, HSI, in conjunction with ML-based classification, could effectively differentiate TSCC from normal tissues with a sensitivity of 94%, a specificity of 68%, and an AUC of 92%. Moreover, HSI paired with DL-based automated classification models offers potential as an intraoperative tumor margin assessment method, capable of achieving an accuracy of 76%, a specificity of 89%, and a sensitivity of 48%^241^. Beyond HSI, other evaluative techniques such as FLI and ESS, when coupled with ML or DL, have displayed substantial promise in identifying HNSCC margins^181,221^. Recently, an OSCC surgery guidance system was developed, combining high-wavenumber RS and ML to build a tissue classification model and a margin length prediction model. The former could discriminate OSCC from healthy oral tissue with 85% sensitivity and 92% specificity, while the latter could predict margin length with commendable accuracy (a mean difference of −0.17 mm, as compared to histopathology)^242^. Tighe *et al*.^243^ evaluated the predictive capabilities of four ML algorithms (J48, random forest, Naive Bayes classifier, and logistic regression) for a positive margin in HNSCC, finding the Naïve Bayes classifier to be the most suitable for case-mix adjustment (AUC=0.72). Generally, although the current integration of imaging techniques with AI is showing promise, it is still in its early stages and requires sufficiently large patient datasets for training, validation, and testing^190,244^.

Despite the continued exploration and development of multiple technologies, each exhibiting its own unique advantages, all come with their inherent limitations^245–247^. To date, a reliable and routinely deployed real-time assessment technology remains elusive^184,248,249^. It is therefore crucial to meticulously investigate the role of these techniques and their oncological outcomes, to synthesize a comprehensive understanding from the numerous studies conducted to date, and to discern the most effective technique for specific clinical scenarios^250^. Furthermore, the creation of an interdisciplinary expert panel encompassing fields such as radiology, AI, pathology, medicine, and physics is highly encouraged. Such a group could significantly enhance the development and improvement of existing technologies, potentially leading to the creation of innovative evaluation techniques through their collaborative efforts^226^.

## Precise head and neck oncology surgery

As technology continues to advance and living standards improve, the pursuit of precision surgery gains greater emphasis^251^. The execution of such surgical precision heavily relies on intraoperative navigation techniques, utilizing imaging, spectroscopy, and endoscopy, as discussed in the preceding sections^199,205,252,253^. Furthermore, numerous preclinical studies have indicated that the integration of virtual reality and augmented reality – termed as virtual surgery – can significantly enhance preoperative planning, resulting in precise resection and reconstruction in complex HNC cases^254–256^. In relation to the resection planning of invaded bone, virtual surgery demonstrates the capacity to accurately delineate and predict the bone margin of HNC^257,258^.

Robot-assisted surgery represents another significant tool in the realm of precision surgery. Its advantages include a wider 3D magnified view, the capacity to visualize ‘around the corner’, and finely controlled movements facilitated by flexible robotic arms and a tremor-filtering system^259,260^. Compared to traditional open treatment, transoral robotic surgery offers a minimally invasive approach, expediting patient recovery and improving aesthetic and functional preservation^261–263^. However, transoral robotic surgery can be more time-consuming than conventional surgery, necessitates a substantial initial investment, and presents a steep learning curve for surgeons^264^. Although it offers a solution to the constrained operative field in the oral cavity, its effectiveness warrants further validation through high-quality observational studies^265–267^. Alongside approved systems like DaVinci and FLEX, several other robotic systems such as MicroRALP, Robo-ELF Scope, and SPORT systems are under development or preclinical testing^268–270^. It is anticipated that future robotic systems will incorporate features like augmented reality and hyperspectral vision and will be characterized by improved visualization, enhanced flexibility, cost-effectiveness, and miniaturization^271,272^.

However, precision in resection should not be misconstrued as minimal radical resection. In certain instances, larger surgical ranges may be necessary for the effective repair of defects in the head and neck region. In the reconstruction process of skin defects, for example, surgical considerations should aim to camouflage scars, avoid destruction of adjacent facial aesthetic units, and when necessary, consider reconstructing an entire aesthetic unit rather than just the damaged part to achieve better aesthetic outcomes^273^. This implies that an unwavering pursuit of minimally complete resection may not always be warranted^274^. In this context, Fan *et al*. devised a flap design technique based on anatomical markers for precision subtotal tongue reconstruction, termed the ‘five-points eight-line-segments’ (FIPELS) technique. When juxtaposed with traditional reconstruction, the FIPELS technique has significantly improved swallowing function and cosmetic outcomes (*P*=0.043; *P*=0.017), thereby enabling a reconstruction-oriented surgical resection^275^.

With an escalating focus on anatomical, aesthetic, and functional aspects, an enhanced repertoire of surgical procedures and treatment strategies has been developed to achieve superior results. Undoubtedly, advancements in cancer therapies (such as adjuvant and neoadjuvant treatment), assessment techniques, and surgical equipment are revolutionizing the manner in which HNSCC is excised and reconstructed^276–278^. In general, we advocate the formulation of the most judicious treatment plan by taking into consideration the patient’s postoperative disease control, quality of life, treatment conditions (such as hospital equipment and physician skills), and a comprehensive understanding of safe SMs.

## Clinical trials pertaining to surgical margins

Clinical trials are indispensable for tracking the progression of research on SMs in HNSCC. In pursuit of the most recent developments, we have gathered relevant clinical trials from www.clinicaltrials.gov. All referenced studies were initiated after 2017, indicative of the clinical significance and congruence with current research trends in the exploration of HNSCC margins. These clinical trials relating to margins in HNSCC can be divided into two key categories: those focusing on SMs (Table 4) and those investigating margin assessment techniques (Table 5). We strongly anticipate that the publication of these clinical trials will bolster surgeons’ confidence in achieving optimal resections.

**Table 4:** Clinical trials of surgical margins for head and neck squamous cell carcinoma.

	Title	Cancer	SM/SD	Study type	Primary outcome measures	Purpose	Study start	Status	NCT number
1	Recurrence in Buccinator Muscle Excision With the Skin Versus Without the Skin in Buccal Squamous Cell Carcinoma	Buccal SCC	Buccinator muscle excision with/without the skin	Interventional	LC (1 year)	To evaluate the LC of buccinator muscle excision with/without the skin	December 2017	Unknown^a^	NCT03364166
2	Clinical Study Evaluating the Proper Surgical Safety Margin for Early Stage Oral Tongue Cancers	cT1-2N0 oral SCC	1.5 cm vs. 1.0 cm	Interventional	LC (2 years)	To achieve a proper surgical safety margin	January 18, 2021	Recruiting	NCT04738786
3	T1 Squamous Cell Carcinomas of the Lip	T1 lip SCC	5 mm vs. 10 mm	Observational	DFS (5 years)	To investigate whether there is a significant difference in DFS between 5 and 10 mm SD	April 1, 2021	Active, not recruiting	NCT05610293
4	Single Modality Trans Oral Robotic Surgery for Primary Oropharyngeal Cancer: Exploring the Impact of Surgical Margins on Local Disease Recurrence	Oropharyngeal SCC	–	Observational	Local recurrence-free survival time (2 years)	To explore the impact of SM on local disease recurrence	September 2, 2021	Recruiting	NCT05065086
5	TPExtreme Induced Chemotherapy Followed by Surgery and Radiotherapy in the Oral Carcinoma	T2-4bN0-3bM0 oral SCC	1–1.5 cm outside the boundary of the residual tumor after IC vs. 1–1.5 cm outside the boundary of the tumor before IC	Observational	Progression-free survival; objective response rate; major partial response (5 years)	To find the best surgery mode after IC	April 1, 2022	Recruiting	NCT05872880
6	Reducing Excision Margins After Neoadjuvant Chemoimmunotherapy for HPV Negative Resectable Locally Advanced HNSCC	Stage III–IVb HNSCC	Reduced SD	Interventional	Disease-free survival (2 years)	To explore the effect of the reduced SD on survival and quality of life	June 22, 2022	Active, not recruiting	NCT05459415

a Study has passed its completion date and its status has not been verified in more than 2 years.

DFS, disease-free survival; HNSCC, head and neck squamous cell carcinoma; IC, induced chemotherapy; LC, local control; SCC, squamous cell carcinoma; SD, surgical distance; SM, surgical margin.

**Table 5:** Assessment methods of head and neck squamous cell carcinoma margin.

	Title	Cancer	Study type	Primary outcome measures	Purpose	Study start	Status	NCT number
1	Image Guided Surgery for Margin Assessment of Head and Neck Cancer Using Cetuximab-IRDye800CW cONjugate	HNSCC	Interventional	Determine the optimal dose of Cetuximab-IRDye800CW for imaging; Determine a threshold level of fluorescence	To determine the optimal dose of cetuximab-IRDye800CW for imaging and a threshold level of fluorescence	December 16, 2017	Unknown^a^	NCT03134846
2	Toluidine Blue Versus Frozen Sections for Assessment of Tumor Margins in Oral Squamous Cell Carcinoma	OSCC	Observational	Diagnostic accuracy of toluidine blue vs. frozen section for tumor margin assessment	To test the accuracy of toluidine blue in the assessment of intraoperative tumor margin	July 2, 2018	Completed	NCT03554967
3	Ultrasound in Tongue Cancer - a Help to Decide Depth of Invasion and to Improve the Surgical Margin	TSCC, Floor of Mouth SCC	Interventional	Depth of invasion; Intraoperative ultrasound helpful during surgery	To investigate if ultrasound can be helpful in the diagnostic work-up	May 28, 2019	Completed	NCT04059861
4	Spectroscopic Analysis of Tongue Tumor Samples	TSCC	Observational	Tumor cells detection in tongue tumor with SpiderMass technique	To accomplish this first phase of machine learning for squamous cell carcinoma of the tongue	October 1, 2021	Recruiting	NCT05104619
5	Gross Examinations Versus Frozen Section for Assessment of Surgical Margins in Oral Cancers	OSCC	Interventional	2-year local recurrence-free survival	To investigate if gross examination and subsequent revision of margin is an equally effective substitute to frozen section based revision	November 15, 2021	Recruiting	NCT04809324
6	Virtual Reality 3D-Surgery Modeling to Enhance Head and Neck Cancer Surgery Quality	HNSCC	Interventional	Physician task load burden; Robotic surgical difficulty	To assess the potential for preoperative virtual reality and 3D pathological modeling	February 4, 2022	Recruiting	NCT05031910
7	Fluorescence-guided Surgery Using cRGD-ZW800-1 in Oral Cancer	OSCC	Interventional	(Highest) mean Tumor-to-background ratio; Rate of adequate (i.e. >5 mm clear) tumor resection margins	To investigate the feasibility of intraoperative fluorescence imaging to adequately assess tumor margins	July 12, 2022	Recruiting	NCT04191460
8	Surgical Margin Assessment by 3D Ultrasound	TSCC	Interventional	Correlation of the SM measurements of the 3D ultrasound to histopathology; Diagnostic accuracy	To investigate the correlation of 3D ultrasound to histopathology to assess tumor margin status	January 1, 2023	Recruiting	NCT05740774
9	Real-time Margin Assessment in Head and Neck Cancer	HNSCC	Interventional	Enhanced intraoperative margin detection	To investigate if the combination of fresh frozen sectioning based on cetuximab-800CW can enhance tumor-positive margin detection intra-operatively	January 1, 2023	Not yet recruiting	NCT05499065
10	3D Ultrasound, Specimen Examination by Surgeon, and MRI in Surgical Margin Assessment	HNSCC	Interventional	Accuracy of the 3D ultrasound and MRI in measurements of the SM; Diagnostic accuracy	To investigate the clinical benefit of 3D ultrasound imaging of the SM	May 15, 2023	Not yet recruiting	NCT05843032

Data availability is not applicable to this review article as no new data were created or analyzed in this study.

a Study has passed its completion date and its status has not been verified in more than 2 years.

3D, three-dimensional; HNSCC, head and neck squamous cell carcinoma; OSCC, oral squamous cell carcinoma; SCC, squamous cell carcinoma; SM, surgical margin; TSCC, tongue squamous cell carcinoma.

## Conclusions and future perspectives

The region of the head and neck is of paramount importance due to its aesthetic and functional roles, and its richness in vital blood vessels, nerves, and organs. Therefore, surgical treatment must ensure tumor-free resection coupled with maximal functional preservation to achieve favorable patient outcomes. Among various prognostic factors, the SM is the one factor within substantial control of the surgeon, thereby necessitating ongoing, rigorous research.

In sum, the minimal safe SD for HNSCC remains an active topic of debate. Current general guidelines suggest an HSD of at least 5 mm, and a CSD of at least 10–15 mm^20^. However, multiple retrospective studies report variable cut-off values for HSD, and it is crucial to note that a clear margin should not be the sole end-goal. The specific characteristics of the tumor, along with the patient’s postoperative quality of life, among other factors, should be judiciously considered in surgical planning. The delineation of the safe SMs is heavily reliant on the progression of margin assessment techniques. The standard FSA technique can no longer satisfy our quest for precise resection. As our understanding of the molecular biology of HSNCC deepens, novel or enhanced imaging techniques will likely emerge, promising significant potential for clinical evaluation. In the era of precision surgery, these imaging techniques serve as surgical navigational tools, and when combined with robot-assisted surgical systems, can guide surgeons toward more minimally invasive and accurate procedures.

Future research should consider several key areas. Firstly, given the current non-uniform classification criteria concerning SMs, there is a pressing need to standardize the fundamental concepts of margins and further quantify tissue shrinkage^279^. Designing additional trials to validate or enhance the clinical feasibility of FSA and other extant assessment techniques is equally worthwhile. Collaborations with experts across various fields to investigate novel assessment techniques are also encouraged. The integration of imaging technology, nanomaterial, AI, and robotic systems promises to refine devices for more precise excisions. It is our sincere hope that an increase in multicenter prospective clinical trials exploring HNSCC margins will ensue, resulting in more satisfactory functional and aesthetic recovery for patients.

## Ethical approval

Ethical review and approval were waived for this study due to the article being a review.

## Consent

Not applicable.

## Sources of funding

This study was supported by the Postdoctoral Science Foundation of China (2018M630883 and 2019T120688), Hubei Province Chinese Medicine Research Project (ZY2023Q015), Natural Science Foundation of Hubei Province (2023AFB665), Medical Young Talents Program of Hubei Province, and Wuhan Young Medical Talents Training Project to L.-L. Bu.

## Author contribution

Y.C., N.-N.Z., and L.-M.C.: conceptualization, investigation, writing – original draft, and visualization; B.L.: investigation, validation, writing – review and editing, and supervision; L.-L.B.: conceptualization, investigation, writing – review and editing, project administration, and funding acquisition. All authors have reviewed and approved the final version of this manuscript for publication. Each author agrees to be accountable for all aspects of the work to ensure that questions related to the accuracy or integrity of any part of the work are appropriately investigated and resolved.

## Conflicts of interest disclosure

There are no conflicts of interest.

## Research registration unique identifying number (UIN)

Not applicable.

## Guarantor

Lin-Lin Bu.

## Data availability statement

The data in this review are not sensitive in nature and are accessible in the public domain. The data are therefore available and not of a confidential nature.

## Provenance and peer review

Not commissioned, externally peer-reviewed.
